# Connection of the SUMO Microscopic Traffic Simulator and the Unity 3D Game Engine to Evaluate V2X Communication-Based Systems

**DOI:** 10.3390/s18124399

**Published:** 2018-12-12

**Authors:** Cristina Olaverri-Monreal, Javier Errea-Moreno, Alberto Díaz-Álvarez, Carlos Biurrun-Quel, Luis Serrano-Arriezu, Markus Kuba

**Affiliations:** 1 Chair for Sustainable Transport Logistics 4.0, Johannes Kepler University, 4040 Linz, Austria; 2 Eurecom, Campus SophiaTech, 06410 Biot, France; errea@eurecom.fr; 3 Instituto Universitario de Investigación del Automóvil (INSIA), Universidad Politécnica de Madrid, 28031 Madrid, Spain; alberto.diaz@upm.es; 4 Institute of Smart Cities; Electrical, Electronic and Communication Engineering, Universidad Pública de Navarra, 31006 Pamplona, Spain; carlos.biurrun@unavarra.es (C.B.-Q.); lserrano@unavarra.es (L.S.-A.); 5 Applied Mathematics and Physics, University of Applied Sciences Technikum Wien, 1200 Wien, Austria; kuba@technikum-wien.at

**Keywords:** traffic light assistance, vehicle-to-everything, traffic simulation, driver-centric simulation

## Abstract

In-vehicle applications that are based on Vehicle-to-Everything (V2X) communication technologies need to be evaluated under lab-controlled conditions before performing field tests. The need for a tailored platform to perform specific research on the cooperative Advanced Driving Assistance System (ADAS) to assess the effect on driver behavior and driving performance motivated the development of a driver-centric traffic simulator that is built over a 3D graphics engine. The engine creates a driving situation as it communicates with a traffic simulator as a means to simulate real-life traffic scenarios. The TraCI as a Service (TraaS) library was implemented to perform the interaction between the driver-controlled vehicle and the Simulation of Urban MObility (SUMO). An extension of a previous version, this work improves simulation performance and realism by reducing computational demand and integrating a tailored scenario with the ADAS to be tested. The usability of the implemented simulation platform was evaluated by means of an experiment related to the efficiency of a Traffic Light Assistant (TLA), showing the analysis of the answer that 80% of the participants were satisfied with the simulator and the TLA system implemented.

## 1. Introduction

Human error as a result of aggressive, intoxicated, drowsy, or distracted driving remains a leading cause of road accidents, substantially affecting road safety [1]. According to the preliminary data from the National Safety Council (NSN) of the U.S, in 2016, 40,000 people died in motor vehicle crashes, an increase of 6% and 14% from the previous years 2015 and 2014, respectively [2]. Additionally, the Federal Highway Administration (FHA) of the U.S. Department of Transportation (DOT) reports approximately 2.5 million intersection accidents annually, which is the second largest category of accidents nationally after rear-end collisions [3,4]. To reduce the amount of accidents and increase safety on roads, cooperative systems that adhere to Vehicle-to-Vehicle (V2V) or Vehicle-to-Infrastructure (V2I) communication (together V2X) use data collected by sensors located in other vehicles or infrastructure to assist the driver [1]. Because these technologies facilitate vehicle-to-vehicle communication relying on Vehicular Ad-Hoc Networks (VANETs) or the transfer of data between vehicles and roadside units, they enable the development of new applications to increase the efficiency of transport networks.

These systems may also unfortunately prove to be a great distraction for the driver, making the testing and assessment phase of such technologies absolutely imperative. This also puts great emphasis on the importance of the use of traffic simulators as an integral tool to test said technologies.

There are different tools in the current state of the art that allow working on different driving aspects separately. For example, micro-simulators like Simulation of Urban MObility (SUMO) or Multi Agent Transport Simulation (MatSIM) [5] allow the generation of realistic traffic environments, which are extended with other works like Vehicles in Network Simulation (VEINS) [6] for intra-vehicular communication or simulators like the Open Racing Car Simulator (TORCS) [7] for driving. However, the existing tools do not cover all the needs in a single solution that allows (i) immersion in first person, (ii) immersion in a virtual traffic environment, and (iii) in turn the extension of different types of tools, including ADAS. More modern environments such as the open-source simulator for autonomous driving research CARLA [8] allow the extension, but to date, it has become complex to create scenarios quickly and manage large volumes of traffic. At the present time, few driver-centric platforms exist in the capacity of allowing realistic modeling of vehicular locomotion after actual traffic models. Thus, the lack of open source, adaptable tools to aid research on ADAS fueled the development of a platform that was both low cost and flexible enough to investigate the effect of V2X communication on a driver’s response through the linking of a 3D game engine-based simulator with a traffic simulator. A 3D driver-centric simulator whose capabilities boast the use of real-world road maps paired with actual traffic models, developed by using Traffic Control Interface (TraCI) protocols, was modeled in [9] as the foundation for the Simulator for Cooperative ADAS and Automated Vehicles (3DCoAutoSim) [10], for which the 3D Driving Simulator with VANET Capabilities to Assess Cooperative Systems (3DSimVanet) [11] and the work presented here are integral parts.

We extend the approach that focuses on simulation performance improvement by reducing computational demand. Additionally, we improve the physics of the steering wheel and integrate a section of the city of Vienna as the urban scenario. As a use case to evaluate the platform, we also implement a TLA.

Our simulation tool generates traffic to provide a driving scenario as a key component of the development in driver-centric simulators based on communication between Unity 3D [12] and the microscopic traffic simulator SUMO [13]. The combination of these two technologies generates an extremely realistic traffic flow structured upon reliable, complex traffic models and the inclusion of a 3D game engine, which allows for the construction of a 3D scenario by providing a mature scripting and physics engine, as well as modularity and scalability, among other characteristics [11].

SUMO was deemed the most worthy and appropriate simulator for our purposes for a variety of reasons, but primarily due to its open-source ability and a tried and proven track record with other projects, such as the VEhicular NeTwork Open Simulator (VENTOS) [14] or VEINS [6], particularly suited to the study of ADAS and vehicular networks. The TraCI protocol was also implemented as a means to retrieve data regarding the number of vehicles, their individual data (i.e., speed and position), and road shape. Our implementation is built over a 3D graphics engine, which serves the purpose of generating the driving scenario through the TraaS library and performs the interaction between the User-Controlled Vehicle (UCV) and SUMO. The resulting simulator’s performance is evaluated through a use case with traffic lights.

The following section provides related literature regarding TLA systems, simulation platforms and their combination. Section 3, Section 4 and Section 5 present the process of implementing the platform, addressing performance improvement and physics, as well as the connection using the TraCI protocol and the integration of scenarios. The retrieval of traffic lights is described in Section 6. A qualitative evaluation of the simulation platform from the driver’s perspective is provided in Section 7. See Section 8 for conclusions and future lines of work.

## 2. Related Work

Evaluation of new in-vehicle technology using simulators has been performed in different works. For example, usability aspects of Head-Up Displays (HUDs) in forward collision warning systems in [15], and a user interface for a novel traffic regulation system in [16]. In this last paper, the interface conveyed information to the driver that was based on the ubiquitous optimized management of individual intersections where physical devices were replaced by Virtual Traffic Lights (VTLs).

### 2.1. Traffic Light Systems

In the same context of traffic lights, traffic signals are unique from one intersection to another. Drivers are not aware of the timing of traffic signals, which causes uncertainty when approaching a traffic light. In particular, electronic traffic signal systems that wirelessly broadcast information about traffic lights can augment the Field Of View (FOV) of the driver even under low visibility conditions [17]. Furthermore, driving patterns such as speed and acceleration are decisive in the estimation of emissions and vehicle fuel consumption [18,19,20]. Therefore, systems that assist the driver by fostering smooth acceleration and deceleration patterns such as TLAs contribute to lower emissions and fuel consumption.

These systems provide essential information to the driver to enhance the visibility of the signals, contributing thus to a decrease of the number of crashes due to intersection conflicts caused by distraction or lack of awareness of the traffic light program. TLA systems use database information to provide environmental real-time data about traffic lights. Based on these real-time data, the algorithms are able to detect traffic lights and calculate the speed that is required from part of the vehicle to reach the traffic light during its green phase. In this work, we test the performance of the developed simulator by means of a TLA use case.

### 2.2. Green Light Optimal Speed Advisory

TLA systems make use of the Green Light Optimal Speed Advisory (GLOSA) algorithm to evaluate the data collected from the traffic lights and calculate the time-to-green. In [21], the V2X Simulation Runtime Infrastructure VSimRTI [22] was used for implementing GLOSA in a realistic scenario. Once the vehicle had entered the communication range of the traffic light, the Road Side Unit (RSU) attached to the traffic light intermittently broadcast Cooperative Awareness Messages (CAMs) including the position timing information and any further pertinent data as it relates to the traffic light. Later, the On-Board Unit (OBU) attached to the vehicle received these messages. Finally, the algorithm was computed by the system, and the driver received a notification with the speed limit within a range. The input values needed for the computation of the algorithm were:

- vehicle’s speed
- vehicle’s acceleration
- vehicle’s distance to the traffic light

### 2.3. Combined Traffic and Network Simulators

Several platforms that simulate V2X have been developed in recent works. Some example simulation models are described in [23]. The authors in [24] combined the network simulator ns-2 [25] with SUMO [13] to evaluate VANETs and developed a TraCI in which SUMO and ns-2 communicated over a Transmission Control Protocol (TCP) to simulate V2V connections. The integrated platform that links SUMO and ns-2 by using the TraCI interface is known as Traffic and Network Simulation (TRANS) [26]. TRANS can mimic traffic congestion and road collisions at a specific location or vehicle.

SUMO was also integrated with the network simulator OMNeT++ in a further work to evaluate the Inter-Vehicle Communication (IVC) protocol [6]. In the same line of research, a communication module to integrate ns-2 and the Simulation of Car-to-Car Messaging (CARISMA) developed by BMW was implemented to simulate and evaluate V2V communication scenarios [27]. The authors used a TCP connection for the simulators and shared information about the number of vehicles and their location in the network at predefined intervals.

### 2.4. Driver-Centric Simulators

The implementation of various simulation platforms from a driver-centric perspective has been presented in several works. For example, the vehicular ad-hoc network In-Car Ergonomics Evaluation Platform (IC-DEEP) is an implementation approach in the form of a Serious Game (SG) that was developed to evaluate the factors that can possibly jeopardize and hamper driving performance as information is manipulated or received from In-Vehicle Information Systems (IVISs) [28,29]. An updated and improved framework that was constructed upon modular components was implemented in an effort to configure new experiments efficiently and facilitate the upgrading process, which also included updating the experiment scenario and conditions [30].

Unity 3D was used in a further work to create a virtual reality system that included vehicles, various weather conditions, and sudden events occluded by blind spots [31].

In the same line of research, the authors in [32] described a concept combining a traffic and driving simulator using Open Street Maps (OSM) [33] and the hardware-accelerated OpenGL renderer based on SUMO, Glosm, to generate the visualization of the three-dimensional module [34].

An interactive motion-based traffic simulation environment was also proposed in [35], where the authors implemented a system involving multiple types of simulation, including driving vehicles such as cars, motorbikes, bicycles, as well as pedestrians.

For a further example of a driver-centric simulator, please refer to [36]. Within this work, SUMO has been used to create traces of vehicles’ movements, which was then utilized by Unity 3D as a means to generate the model traffic, without explicit communication between Unity 3D and SUMO.

Despite the fact that some simulators described within this section provided the benefits of low cost, open source, and flexibility, they simply were unable to fulfill the specific requirements properly without further alteration.

Our contribution to the state of the art is measured by our development of a driver-centric simulation that is built over a 3D graphics engine. This engine has the ability to generate simultaneously the driving scenario and communicate dynamically with a traffic simulator as a means to replicate real traffic conditions. We applied the TraaS library to perform the interaction between SUMO and the user-controlled vehicle.

## 3. Simulation Implementation

**Figure 1:**
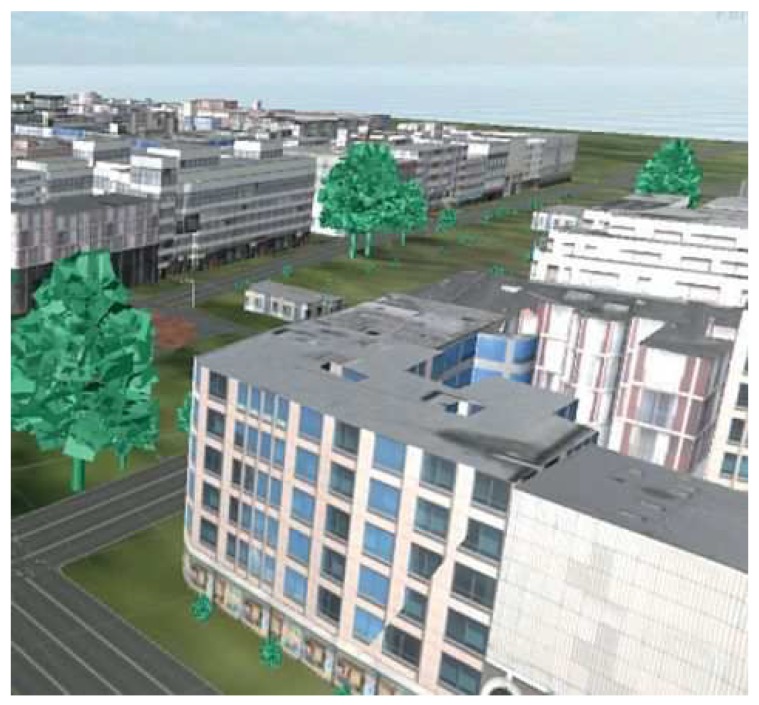
Implemented urban scenario relying on the CityEngine modeling tool, OpenStreetMap data, and the GeoTIFF data available from the city of Vienna.

For the extension of the simulation platform, we selected as the scenario the Vienna neighborhood “Neubaugurtel”. To create the urban, customized scenario, we used the CityEngine procedural modeling tool [37], OSM data, and the GeoTIFF data provided by the city of Vienna [38]. Figure 1 depicts the implemented urban scenario resulting from the combination of the data.

We then displayed the scenario in Unity, after having created it in SUMO following the process below:

1. Generation of the road set, which built the network scenario through the process of retrieving all of the vertices, allowing for the definition of lane shape, and created a GameObject per segment. Every segment was noted in meters as is required by the SUMO X-Y coordinate system and then mapped to the X-Z coordinate system in Unity 3D.
2. In accordance with its position and angle, each simulated vehicle generated by SUMO was placed in Unity as a GameObject.

Every vehicle involved in said simulation was given a predetermined path through the scenario. These routes were designed to mimic real-life traffic flows taken from a traffic light scenario in the local area. All vehicle types and their defaults are shown in Table 1.

As each vehicle had a predefined route among the scenarios, it was necessary to update their positions and angles every time step, so that the movement could be displayed in Unity 3D. We implemented the method FixedUpdate() in an effort to make a change of the scene to the following frame, thus making the assurance of a constant number in the Frames-Per-Second (FPS).

A Mini Cooper was incorporated as the UCV’s chosen model, outfitted with the required physics to complete locomotion through the given environment. Given the fact that the simulation is seeking to examine driver behavior and does not pay particular attention to the metrics associated with this interaction with traffic, data collection for speed and position was not recorded even if available.

As previously mentioned, a connection between SUMO and Unity 3D was created via the TraCI protocol. First, Unity 3D sent a connection request to SUMO, and once it was accepted, the behavior of the simulation could be modified from Unity.

**Figure 2:**
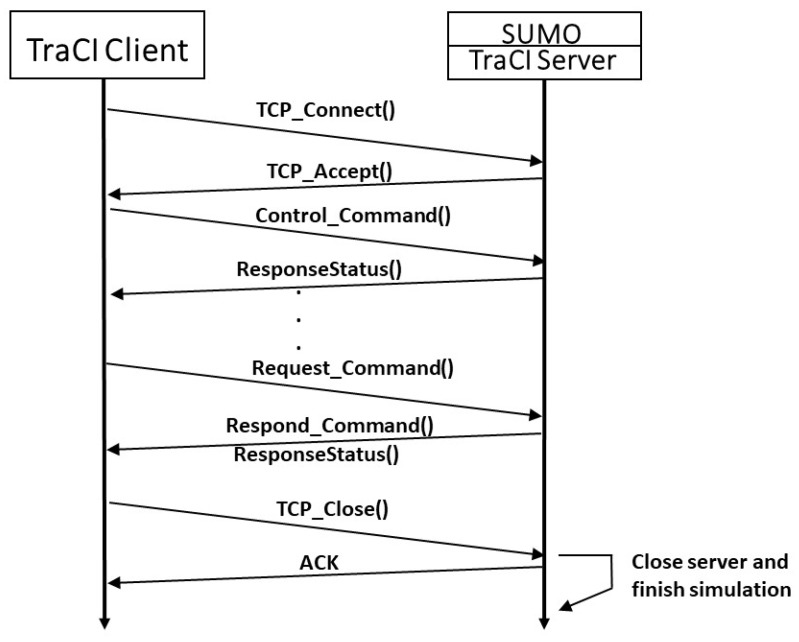
Implemented communication between SUMO and the TraCI client.

TraCI provides the platform for the communication protocol, ensuring control and modification of the simulation process parallel with SUMO. Beyond this, the system also is capable of retrieving any elements within the system that are being implemented in the simulation, such as edges, junctions, traffic lights, or vehicles. Moreover, each of these objects contains variables that can be retrieved or modified online. Figure 2 illustrates the communication between a TraCI client and SUMO. The developed script SumoCon guarantees the connection of both the generation of the scenario and SUMO. Once the scenario had been initialized, the algorithms that will be explained in the following sections were executed in order to retrieve information about the simulated objects of interest.

## 4. Sumo Network and Traffic in Unity 3D

The SumoCon script was in charge of the interactions between SUMO and Unity 3D. It contained a set of functions, each of which was used for different purposes. When the simulation scene was run for the first time, the SUMO simulation was set, and initial SUMO objects’ information was retrieved by Unity. SUMO data were categorized into two groups:

- static, meaning that the information did not change during the simulation;
- dynamic, referring to the information that needed to be updated every simulation step.

### 4.1. Initialization

Algorithm 1 describes the initialization of the simulation. Once the connection was initialized, the algorithm read a file that contained the information regarding the route to follow, as well as the information regarding the lanes, simulated vehicles and traffic light systems.
**Algorithm 1** Initialization.**Result:** Simulator connected with SUMO and scenario loaded1:Connect to SUMO2:ReadLanes()3:ReadVehicles()4:TLSSumoNet(0)

### 4.2. Reading and Displaying SUMO Data

In order to read the data from SUMO, the information about the road network was retrieved as shown in Algorithm 2. A list with all the existing lanes in the road network was extracted from SUMO. Each component of the list was defined as a Lane object in SUMO and was characterized by a set of parameters containing information regarding the lane’s position, length, shape, etc. Out of all these available parameters, shape, width, length, and allowed vehicles were used to define the lane in Unity 3D.

**Algorithm 2** Read lanes.**Result:** List of links l
1:$l_{s}\leftarrow$ Retrieve all lanes from SUMO2:l=∅3:**for all** lanes $l_{s}\in l_{s}$ **do**4:    v← Retrieve $l_{s}$ vertex from SUMO5:    w,h← Retrieve $l_{s}$ (width, height) from SUMO6:    type← Retrieve $l_{s}$ type from SUMO7:    l←l∪{(v,w,h,type)}8:**end for**9:DisplayLanes()

After defining these parameters, each object of the type Lane was stored in a collection of the type Link. The Link class was formed by several methods to reference objects of the type Link. Then, the information about SUMO roads was used to print the network as a GameObject. The data regarding the roads were considered static, so the algorithm was used once.

Once the information related to the road network was read, Algorithm 3 was called in order to display every lane as a GameObject in Unity 3D.

**Algorithm 3** Display lanes.**Input:** List of links **l** **Result:** Display all lanes in the scenario1:**for all** lanes l∈l **do**2:    **if** $l_{type}\in \left\{\mathrm{car}\right\}$ **then**3:        v0xv0y←first(lv)4:        **for all** vertex vixviy∈tail(lv) **do**5:           l=∥vixviy−v0xv0y∥26:           $c=(\frac{v_{0_{x}}+v_{i_{x}}}{2},\frac{v_{0_{y}}+v_{i_{y}}}{2})$7:           $\theta =\arctan(\frac{x-x0)}{\left(z-z0\right)}*\frac{180}{\pi })$8:           DrawLane(l,c,θ)9:           v0xv0y←vixviy10:        **end for**11:    **end if**12:**end for**

To print the lanes in Unity, every object contained in the Link list that was created in Algorithm 2 needed to be instantiated.

As previously mentioned, every lane can be defined by its shape, length, and angle with respect to the coordinates system. For example, shape contains indirect information about the position of the lane as it is formed by a set of *x*, *y* points.

A new GameObject is created and instantiated for each lane. If the lane id of the next lane to print was Start_1, then the UCV was instantiated to be the beginning point of the route that the user had to drive through.

Once the network was printed, a similar algorithm was used for reading and printing SUMO simulated traffic. The main difference is that these data are considered dynamic as the traffic is constantly moving. Therefore, the methods used for the simulated cars needed to be called every SUMO time step. Algorithm 4 retrieves a list containing the speed, angle, and position of every simulated vehicle from SUMO. Then, an object of the type Auto is created for each simulated vehicle and stored in a list.

Finally, Algorithm 5 iterates the Auto list generated by Algorithm 4 and generates a new GameObject for each vehicle in the list, depending on the configured departure times of the rou.xml file. The file contains all the predefined routes of the traffic generated by SUMO, i.e., all the points through which the car generated in SUMO will drive.

The vehicle is then instantiated in the scenario only if the distance to the UCV is smaller than 60 m, as explained in the next section. In addition, if a vehicle already existed in the simulation, this function updates its variables. The movement of the simulated cars is performed with the method move(), which updates the position and orientation of the simulated vehicles every time step.

**Algorithm 4** Read vehicles.**Result:**
List of simulated vehicle data v1:arrived ← Last step added vehicles from SUMO2:left ← Last step removed vehicles from SUMO3:v←(v∪a)∩d4:DisplayVehicles()

**Algorithm 5** Display vehicles.**Input:***u*, **v**, **s**, distance *m* **Result:** Display vehicles with distance ≤m
1:DisplayVehicles(u,v,d)2:**for all** vehicles v∈v **do**3:    d←∥vxvy−uxuy∥24:    **if** d≤m **then**5:        **if** v∈s **then**6:           Actualize coordinates of *v*7:        **else**8:              Update list: s←s∪{v}9:        **end if**10:    **else**   Update list: s←s∖{v}11:    **end if**12:**end for**

## 5. Simulation Performance Improvement

This paper extended a version that proved to be exceptionally demanding in terms of computation to perform a request of the vehicles in the network on every frame and also resulted in movements that were unrealistic. To improve simulation performance and address these issues, instead of reading and displaying every vehicle contained in the network, only the ones within a range of 60 m to the UCV were displayed in Unity 3D (see Algorithm 5). This update successfully increased the performance of the simulation. In the algorithm, we denote by *u* the user-controlled vehicle. The lists v and s contain all other vehicles and all currently displayed vehicles.

In the previous version, the simulation step that was implemented in SUMO was 100 ms. As a result of this, it appeared as if the vehicles would teleport from one spot to another in every frame.

This behavior was corrected in the current version by increasing the frequency with which the readCars() function was called from the FixedUpdate() and setting it equal to five Time.deltaTime in the Unity 3D scripting API. This coordination of the SUMO simulation timeStep and Unity Time.deltaTime made it possible to considerably improve the movement of the simulated vehicle:

- Unity Time.deltaTime =0.02 s
- 0.02×5=0.1
- SUMO timeStep =0.1 s

In terms of physics related to the sensitivity of the simulation environment, we improved the steering wheel force feedback. This was achieved by synchronizing the steering system with the front wheels. The wheels were grouped as left and right wheels, front and rear (see Algorithm 6).

**Algorithm 6** Wheel.**Input:***δt* **Result:** Update of wheels and steering wheel
1:UpdateSteering()2:**for all** Wheels *w* **do**3:    sign ←14:    **if** *w* is a front wheel **then**5:        sign ←−16:    **end if**7:    Rotate *w* by roundsPerMinute(*w*)·sign$\cdot \frac{1}{6}\cdot \delta t$.8:**end for**9:**for all** Front wheels *w* **do**10:    Adjust angle by steering wheel angle11:**end for**12:Adjust steering wheel according to front wheel

## 6. Retrieval of the Traffic Light System

SUMO contains information about Traffic Light Systems (TLSs) instead of individual traffic lights. Due to this fact, traffic lights were placed manually in Unity, programming their timing according to the correspondent SUMO location information.

The .net.xml file generated from the netconvert tool includes information about the TLSs. SUMO defines a fixed-time program for each, and it can be either static or actuated.

Not all the TLS programs from junctions with only one intersection are automatically retrieved, including a red phase in the .net.xml file. Sometimes, the phase duration does not correspond with reality either. For this reason, missing red phases were added or inaccurate information regarding the duration was corrected in the TLS programs. Once these two modifications were performed, the edition of the network was completed.

In order to place the traffic lights in Unity, an asset called “Traffic Lights” was used. Then, a C# script was created to make the animation of the traffic lights, as well as to set the timing program equal to SUMO.

**Figure 3:**
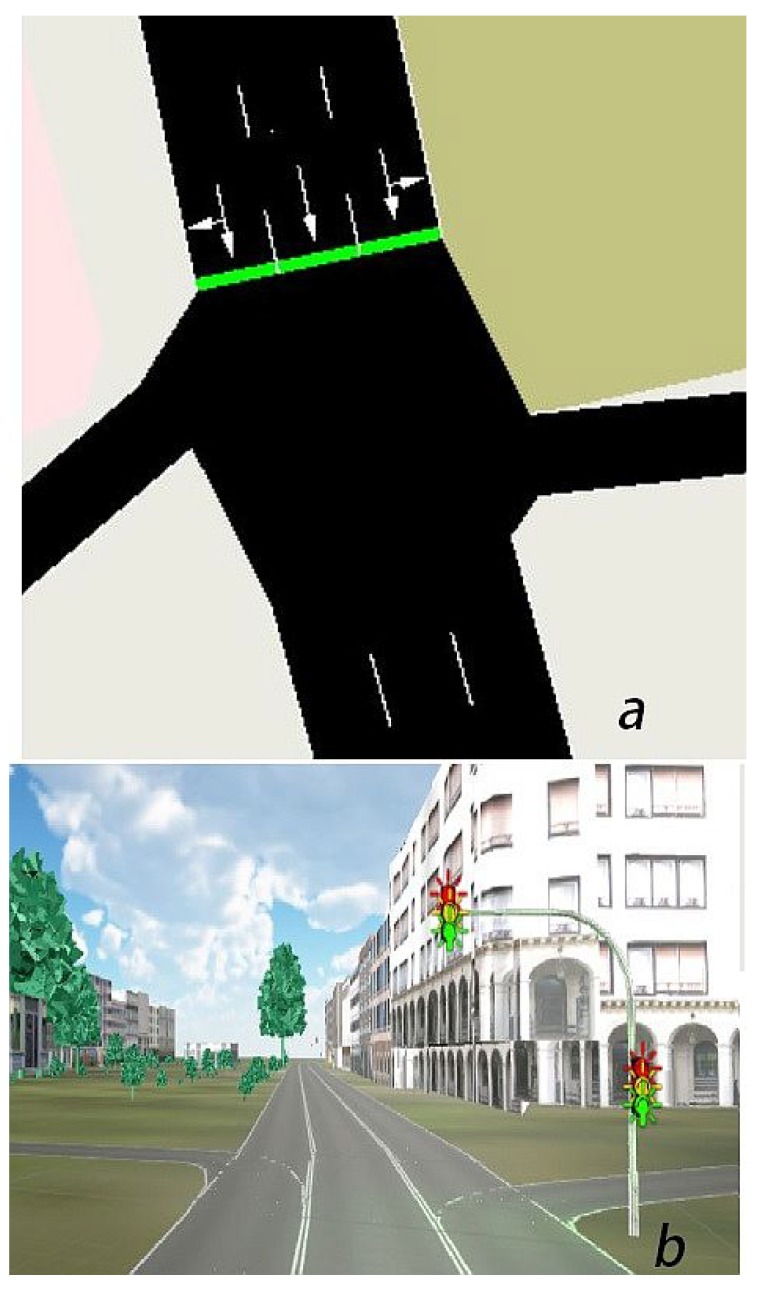
(**a**) Traffic light system in SUMO that was replicated as individual traffic lights in Unity (**b**).

Figure 3 depicts the process. In order to illustrate the retrieval of the TLS, we focus on a use case for which we implemented a traffic light assistant.

Algorithm 7 read all the TLS from the SUMO network and stored them in a list in the same manner as vehicles and lanes. The current simulation time determines the absolute time until the next phase change. The time function is called every SUMO simulation step in order to update the information of the TLS.

Each TLS is stored in a list so that its information can be used anytime during the simulation. To make the state of the information accessible, getter methods are created for each of the variables retrieved from the TLS as described below:

- getPhase(id): gets the actual phase of the traffic light.
- getNextSwitch(id): gets the time left until the next change of phase. The current simulation time needs to be subtracted from this time in order to obtain the relative next switch time.
- getRedYellowGreenState(id): gets the actual state of the TLS. Each character of the string represents each of the links that the TLS controls.
- getPhaseDuration(id): gets the total time of the current phase.
- getControlledLinks(id): returns a list with the following structure: from[] to[] through[]. It contains information about from which lane we come (from), to which lane we are going next if we are in the current lane (to) and through which lane, junction we will reach the future lane to which we are going (through).
- getCompleteRedYellowGreen(id): returns a list with the complete program of the TLS; total number of phases, duration of each phase, and state of each phase.

In addition to the process described to retrieve the traffic lights from SUMO and display them in Unity 3D, Algorithm 8 computes whether a traffic light is located at the end of the current lane of the UCV. *ℓ* refers to the list of links and *c* to the current lane. The function returns either a string with the traffic light ID or a null value. If the returnValue is not null, then further information related to the UCV, such as speed and distance to the end of the lane from its current position, will be retrieved. This is necessary in order to be able to implement the traffic light assistant using a calculation of the estimated time that the vehicle will need to reach the traffic light.

In the following, t refers to the list of traffic lights and *c* to the current lane. Each traffic light t∈t.

**Algorithm 7** TLS SUMO Net.**Input:** Simulation time *t* **Result:** All the TLSs status updated1:$t_{s}\leftarrow$ Retrieve all TLSs from SUMO2:t=∅3:**for all** SUMO traffic lights $t_{s}\in t_{s}$ **do**4:    $t_{\mathrm{left}}\leftarrow$ NextSwitchPhase$(t_{s})-t$5:    $t=\leftarrow t\cup \{t_{s},t_{\mathrm{left}}\}$6:**end for**

**Algorithm 8** Traffic light.**Input:** c, **t** **Result:** String containing Information about traffic lights1:TrafficLightAtLane(c,t)2:Initialize String *r*3:**for all** traffic lights t∈t **do**4:    Access list of links ℓ(t)5:    **for all** l∈ℓ(t) **do**6:        **if** Starting Position from(l) equals *c* **then**7:           $r\leftarrow t_{\mathrm{id}}$8:        **end if**9:    **end for**10:**end for**11:**return***r*

A comparison between the current lane and the lanes controlled by each TLS is performed. If they match, the TLS ID is stored and returned to the main procedure.

As previously mentioned, we implemented a TLA system as a use case to test the linking of SUMO and Unity 3D within the simulation platform. The system relies on the GLOSA algorithm to determine the time until the next switch based on the remaining duration of the current phase.

We verified the distance of the vehicle to the traffic light by getting the position of the UCV and the traffic light at each simulation step (see Algorithm 9).

If the distance returned by the algorithm is smaller than 80 m, the GLOSA algorithm is computed, and several messages are displayed on the dashboard screen depending on the result of the algorithm as follows:

- “Actual speed OK”. The system tells the driver that the actual speed is optimal to approach the next intersection.
- “Slow down to X km/h”. The systems warns the driver that the speed needs to be decreased in order to approach the next intersection correctly.
- “Optimal speed between X-Y km/h”. The systems warns the driver that the speed needs to be increased to the range of speeds displayed.
- “Stop at line”. The system warns the driver to stop at the traffic light due to the long duration of the red phase and due to the low speed calculated by the algorithm.

**Figure 4:**
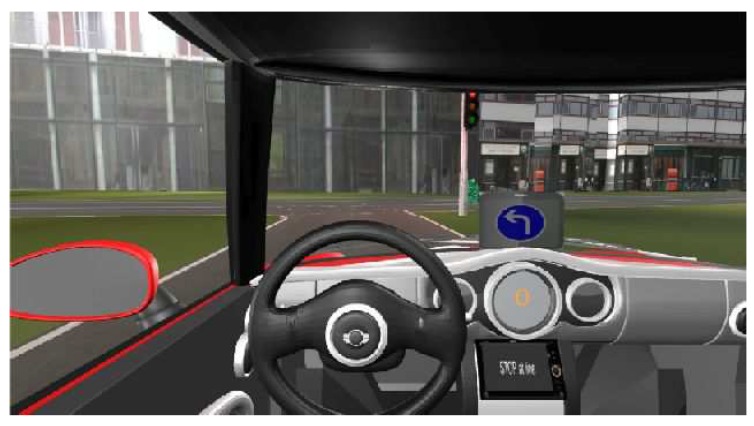
Warning message displayed in the cockpit according to the current speed compared to the optimal one [39].

Figure 4 shows one example for a message displayed in the cockpit according to the current speed compared to the optimal one.

**Algorithm 9** Distance UCV to traffic light.**Input:***u*, c, *ℓ* **Result:** Distance *d* to the end of the lane from current position1:DistanceUCVToTrafficLight(c,ℓ)2:**for all**l∈ℓ**do**3:    **if** $l_{id}$ equals *c* **then**4:        Point P←lastPointOf(l)5:    **end if**6:**end for**7:Calculate Euclidean distance d←∥PxPy−uxuy∥28:**return***d*

TLSs programs provide a behavioral description of the traffic lights that are located at intersections. The complete program returns a list with the information of all the states of the traffic light and their duration. For example, in the following state definition of the TLS phase, <phaseduration = “36” state=GGrrrrrGg/>, each character in the string within a phase’s state describes the state of one link of the TLS (g for green, y for yellow, r for red).

Once the information of the UCV was obtained, the retrieval of the traffic light program information was performed (time until next switch, actual state g (green), y (yellow), or r (red), controlled links, and complete program). We retrieved the index of the actual state inside the list of the complete program, the character (from the state string) corresponding to the current lane of the UCV, and finally, the complete time until it was green again (if the traffic light was in the yellow or red state). Algorithm 10 describes the steps for the retrieval of the relevant information.

**Algorithm 10** Time to next switch.**Input:** Closest TLS *tls* **Result:** An interval $\left[t_{\min},t_{\max}\right]$ (see Algorithm  11)
1:$tls_{\mathrm{desc}}\leftarrow \emptyset$2:**for all** Status s∈tls **do**3:    $tls_{\mathrm{desc}}\leftarrow tls_{\mathrm{desc}}\cup \left\{\mathrm{State}\left(s\right),\mathrm{Duration}\left(s\right)\right\}$4:**end for**5:**return** TMinTMax$\left(\mathrm{Lane}\left(u\right),tls_{\mathrm{desc}}\right)$

Algorithm 11 calculates the exact time t until the next green light phase. tmin represents the time at which the next green light starts, and tmax represents the time at which the next green light will end.

**Algorithm 11** Time min max.**Input:** Lane *l*, *tls*_desc_ **Result:** Time until next switch $t_{\min}$, time until next “green” state $t_{\max}$
1:$tls_{s}\leftarrow$ CurrentState$\left(tls_{\mathrm{desc}}\right)$2:$t_{\min}\leftarrow \mathrm{Sec}\mathrm{ondsToNextSwitch}\left(tls_{s},tls_{\mathrm{desc}}\right)$3:$t_{\max}\leftarrow t_{\min}$4:**if**$tls_{s}$ in green state **then**5:    $tls_{s}\leftarrow \mathrm{NextState}\left(tls_{s},tls_{\mathrm{desc}}\right)$6:**end if**7:**while**$tls_{s}$ not in green state **do**8:    $t_{\max}\leftarrow \mathrm{Sec}\mathrm{ondsToNextSwitch}\left(tls_{s},tls_{\mathrm{desc}}\right)$9:    $tls_{s}\leftarrow \mathrm{NextState}\left(tls_{s},tls_{\mathrm{desc}}\right)$10:**end while**11:**return**$\left(t_{\min},t_{\max}\right)$

After having stored all the information needed in different variables, the next step was the computation of the GLOSA algorithm that compared the time to the end of the lane (for the user with its actual speed) with the range of times obtained (tmin-tmax) depending on the state of the traffic light. The system calculated the speed needed to be able to arrive at the next traffic light in green. The code of the algorithm is shown in Algorithm 12. The function returns true if the actual speed is adequate or false if the actual speed needs to be modified.

Finally, the code of the Algorithm 13 displays the optimal speed to reach the traffic light during its green phase by recommending that the driver maintain the current velocity, accelerate, or decelerate.

**Algorithm 12** Time to end of lane.**Input:***t*_min_, *t*_max_, time to reach TLS *u_t_*, actual speed *u_s_*, distance to next TLS *d* max speed allowed *v_M_* **Result:**
$v_{\min},v_{\max}$ in case a speed change is necessary or ∅ otherwise
1:**if** not $t_{\min}\leq u_{t}\leq t_{\max}$ **then**2:    $v_{\min}\leftarrow \left[\frac{d}{t_{\min}}*3.6\right]$3:    **if** $u_{t}>t_{\max}$ and $v_{\min}\leq v_{M}$ **then**4:        $a_{\mathrm{needed}}=2*\left(d-\frac{u_{s}*t_{\max}}{3.6}/t_{\max}^{2}\right)$5:        $a_{\max}\leftarrow$ MaxAccel(u)6:        **if** $a_{\mathrm{needed}}<a_{\max}$ **then**7:           $v_{\max}\leftarrow argmin\left(\left[\frac{d}{t_{\min}}*3.6\right],v_{M}\right)$8:        **else**9:           $v_{\min}\leftarrow v_{\max}\leftarrow 0$10:        **end if**11:    **else**12:        $v_{\max}\leftarrow v_{\min}$13:    **end if**14:    **return** $\left(v_{\min},v_{\max}\right)$15:**elsereturn** None16:**end if**

**Algorithm 13** Visual advise.**Input:***t*_min_, *t*_max_, time to reach TLS *u_t_*, actual speed *u_s_*, distance to next TLS *d* max speed allowed *v_M_* **Result:** A visual advise to the vehicle *u*
1:m← “Actual speed OK”2:vminvmax← TLRAlgorithm$\left(t_{\min},t_{\max},u_{t},u_{s},d,v_{M}\right)$3:**if**vminvmax≠0→**then**4:    **if** $v_{\min}=v_{\max}$ **then**5:        **if** $v_{\min}>u_{s}$ **then**6:           m← “Speed up to $v_{\min}$ km/h”7:        **else**8:           **if** $v_{\min}=0$ **then**9:               m← “STOP at line”10:           **else**11:               m← “Slow down to $v_{\min}$ km/h”12:           **end if**13:        **end if**14:    **else**15:        m← “Optimal speed between $v_{\min}-v_{\max}$ km/h”16:    **end if**17:**end if**18:DisplayMessage(m)

## 7. Platform Evaluation

We asked a total of 25 participants (mean age = 27.5, SD = 9.2, 64% males, 36% females) to evaluate the efficiency of the TLA. After being welcomed, each participant was instructed about the experiment procedure. After 5 min of time to get familiarized with the simulation platform, the participants drove 30 min through the developed urban scenario, half of the time with the TLA activated and half of the time without the TLA.

The route to be followed was conveyed to the drivers by using arrows in an in-vehicle display. A TLA displayed additional information related to the ideal speed in order to arrive at the traffic light while it was green. As the information conveyed was only a recommendation, the visual information was not combined with acoustic signals. The messages were organized according to their critical or informative nature, taking into account their prioritization in a proper location in the vehicle [1].

Once the experiment was finalized, a qualitative questionnaire was completed by the participants. The analysis of the answers showed that 80% of the participants were satisfied with the simulator and the TLA system implemented. The scenario in which they were driving was scored as realistic, and they were also able to recognize the physical surroundings. They found the simulated vehicle characteristics to be realistic as well. Motor sound and advancement of steering wheel physics also contributed to the enhanced perception of realness. Table 2 shows the items of the questionnaire and the results of the analyzed data.

In terms of the in-vehicle messages displayed, 28% of the participants felt overwhelmed by the amount of information to pay attention to (TLA messages, traffic lights, navigation arrows). Twenty percent suggested placing the dashboard screen with TLA messages in a higher position in order to reduce the eyes off road time, and 12% even suggested using an acoustic signal instead of a visual message to convey the information.

## 8. Discussion

This work presented a 3D driver-centric simulator whose capabilities included the ability to pair real-world road networks with realistic traffic models by implementing TraCI protocols. We extended the original work presented in [9,39] and focused on improving simulation performance and realism by reducing the computational demand.

The implementation was built on a highly capable 3D graphics engine that is able to generate the driving scenario as it also communicates with the traffic simulator to create replications of actual traffic conditions.

We included a section of the city of Vienna as an urban scenario and a procedure that only renders vehicles that are within a certain radius from the driver in order to increase the accuracy, efficiency of computation, and ease of configuration between UCV and Non-Player Controlled (NPC) vehicles.

**Figure 5:**
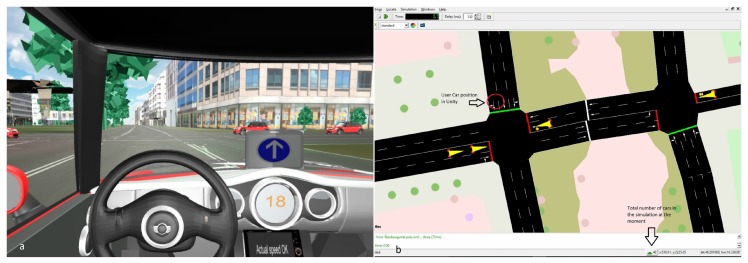
Example of the number of NPC vehicles contained in the Unity (**a**) and SUMO (**b**) scenarios.

In addition, we retrieved information from different objects that are programmed in SUMO such as traffic lights and included them in the 3D scenario by using the TraCI protocol. The gain in computational power allowed for the possibility to increase in the scenario the number of NPC vehicles contained depending on their location within the range of 60 m to the UCV. For example, Figure 5 depicts three vehicles in the simulator despite SUMO having 42 loaded at this time.

Eighty meters was deemed the appropriate retrieval distance of traffic light systems given that the urban, city environment dictates a lower maximum speed and shorter distances in between traffic lights. Drivers also need advanced warning in order to allow for proper reaction to messages transmitted from the vehicle; thus, we estimated that the optimal distance to initialize the algorithm was between 80 and 100 m. As per the results of the simulation, participants reacted and performed well to 80 m, giving us cause to maintain said distance.

With the new release of SUMO that makes it possible to insert the UCV of Unity 3D into SUMO, future work will address updates of relevant UCV data (including position, speed, and heading) to the simulator in order to feasibly measure the impact of the driver behavior on traffic and the inclusion of capabilities in the simulation environment. This will enable the evaluation of applications based on V2V communication and the performance of specific research on VANET-based ADAS. For example, traffic light information can be sent to the first vehicle in a queue that will then be transmitted to the vehicles behind.

In terms of the potential visual distraction and cognitive load generated by interacting with the in-vehicle information, we designed the communication of messages taking into account their critical or informative nature, and consequently their prioritization in a proper location in the vehicle [1]. However, further evaluations should be performed in order to validate the subjective opinions of part of the participants.

## Figures and Tables

**Table 1:** Configuration values for the traffic simulator.

Parameter	Value/s
Max speed	30 ms${}^{-1}$ (although, it depends on the maximum speed of the road)
Acceleration	2.6 ms${}^{-2}$
Deceleration	4.5 ms${}^{-2}$
Dimensions	5 l × 1.8 w × 1.6 h
Minimum gap	2.5 m
Vehicle class	Passenger
Car Following model	Krau**ß** (ss)
Lane Change model	LC2013

**Table 2:** Items of the questionnaire and the correspondent results from the analyzed data (Likert scale range: 1 = strongly disagree to 5 = strongly agree).

Items	Mean Value (SD)
Q1 Messages displayed by the system were clear and intuitive	4.32 (0.73)
Q2 Driver’s behavior was influenced by the TLA while approaching a traffic light	4.24 (0.7)
Q3 The TLA system was not distracting while driving	3.08 (1.01)
Q4 The TLA system was helpful/useful	4.12 (0.81)

## Abbreviations

The following abbreviations are used in this manuscript:

ADAS	Advanced Driving Assistance System
CAM	Cooperative Awareness Message
DOT	U.S. Department of Transportation
FHA	Federal Highway Administration
FOV	Field Of View
GLOSA	Green Light Optimal Speed Advisory
HUD	Head-Up Display
IC-DEEP	Vehicular Ad-Hoc Network
IVC	Inter-Vehicle Communication
IVIS	In-Vehicle Information System
NPC	Non-Player Controlled
NSC	National Safety Council
OBU	On-Board Unit
OSM	Open Street Maps
RSU	Road Side Unit
SG	Serious Game
SUMO	Simulation of Urban Mobility
TCP	Transmission Control Protocol
TLA	Traffic Light Assistant
TLS	Traffic Light System
TraaS	TraCI as a Service
TraCI	Traffic Control Interface
TRANS	Traffic and Network Simulation
UCV	User-Controlled Vehicle
V2I	Vehicle-to-Infrastructure
V2V	Vehicle-to-Vehicle
V2X	Vehicle-to-Everything
VANET	Vehicular Ad-Hoc Network
VTL	Virtual Traffic Light
